# Advances and critical aspects in cancer treatment development using digital twins

**DOI:** 10.1093/bib/bbaf237

**Published:** 2025-06-02

**Authors:** Rym Bouriga, Caroline Bailleux, Jocelyn Gal, Emmanuel Chamorey, Baharia Mograbi, Jean-Michel Hannoun-Levi, Gerard Milano

**Affiliations:** Department of Medical Oncology, Antoine Lacassagne Center, University Côte d’Azur, 33 Avenue de Valombrose, 06189 Nice, France; Department of Medical Oncology, Antoine Lacassagne Center, University Côte d’Azur, 33 Avenue de Valombrose, 06189 Nice, France; Department of Epidemiology and Biostatistics, Antoine Lacassagne Center, University Côte d’Azur, 33 Avenue de Valombrose, 06189 Nice, France; Department of Epidemiology and Biostatistics, Antoine Lacassagne Center, University Côte d’Azur, 33 Avenue de Valombrose, 06189 Nice, France; FHU OncoAge, IHU RespirERA, IRCAN, Inserm, CNRS 7284, U1081, University Côte d’Azur, 06189 Nice, France; Departement of Radiotherapy, Antoine Lacassagne Center, University Côte d’Azur, 33 Avenue de Valombrose, 06189 Nice, France; Scientific Valorization, Antoine Lacassagne Center, University Côte d’Azur, 33 Avenue de Valombrose, 06189 Nice, France

**Keywords:** drug discovery, oncology, artificial intelligence, digital twins, clinical trials

## Abstract

The emergence of digital twins (DTs) in the arena of anticancer treatment echoes the transformative impact of artificial intelligence in drug development. DTs provide dynamic, accessible platforms that may accurately replicate patient and tumor characteristics. The potential of DTs in clinical investigation is particularly compelling. By comparing data from virtual trials with conventional trial results, medical teams can significantly enhance the reliability of their studies. Moreover, a significant breakthrough in clinical research is the ability of DT to augment patient data during ongoing trials, enabling adaptive trial designs and more robust statistical analyses to be performed even with limited patient populations. The development of DTs faces however several technical and methodological challenges. These include their tendency to produce unreliable predictions, non-factual information, reasoning errors, systematic biases, and a lack of interpretability. Future research in this field should focus on an interdisciplinary approach that brings together experts from diverse fields, including mathematicians, biologists, and physicians. This collaborative strategy promises to unlock new frontiers in personalized cancer treatment and medical methodologies.

## Introduction

The conventional approach to performing clinical trials, classically divided into four phases to assess the safety and effectiveness of novel treatments, is fraught with inefficiencies. These trials often span over a decade, incur costs of around $50 000 per participant, and yield a mere 10% success rate from the initial phase onwards [1–3]. Moreover, clinical trials aim to identify an average optimal treatment for broad populations, thus restricting the capacity to customize therapies for individual patients. This limitation is particularly problematic in cancer research, where the diverse nature of tumors leads to significant variations in patient responses to treatment [4].

The strategic importance of modeling and simulation in clinical trials has grown considerably [5]. Of note is the initiative by Creemers and coworkers [6], who proposed a mathematical model application to realize cancer immunotherapy trials *in silico*, a significant advance able to predict survival and response profiles of various treatment regimens. This methodological shift was accompanied by the 2018 congressional mandate encouraging the U.S. FDA to adopt *in silico* approaches to improve R&D outcomes [7, 8]. The adoption of Artificial intelligence (AI) was promising since it has the potential to promote innovative trials and accelerate their development in oncology by acting both at the preclinical level and in the clinical field [9, 10]. The present article explores how digital twin (DT) can reshape biomedical research in oncology by addressing the complex and multifaceted challenges inherent to preclinical and clinical drug development and ultimately paving the way for breakthroughs in personalized medicine. Relative exhaustive reviews covering the domains of new drug development and DT are at disposal [11–14]. Consequently, the present position paper did not aim to specifically intervene in this area. The purpose was herein to help identify the strengths, weaknesses, and limits generated by the interface between DTs and drug development in the anticancer field.

## DTs—definitions and applicability in cancer treatment

DT development and performance represent a disruptive technology built upon various machine learning (ML) architectures [13]. ML can derive models and solve complex problems from large and heterogeneous data sets (biological and/or clinical data). DTs thus consist of generative models initiated by a system’s real biological and physical characteristics at a given time. In healthcare, a DT represents the digital counterpart of the patient, created through learned data distribution with sequential or time-dependent patterns, which have been incorporated into a mathematical representation (model). DTs are distinguished by their dynamic nature, constantly updated through real-time data integration [15]. Consequently, there is a bidirectional flow of information between a given biological entity and its DT, resulting in virtual data influencing decision-making. By functioning as intelligent and adaptive systems, DTs can impact all drug discovery and development stages, from target identification to clinical trials and post-market surveillance [13].

The current research and development activity in cancer remains particularly resource-intensive and encounters a significant failure rate [16]. The high attrition rate in oncology drug development contributes to escalating costs and extended timelines, creating an urgent need for more efficient approaches. Fast-tracking of cancer drug development is thus emerging as a priority, with regulatory agencies implementing various accelerated approval pathways to address unmet medical needs [17]. DTs offer potent solutions in this context by constituting digital human representations that are able to translate their physiological and anatomical characteristics with remarkable fidelity [18]. These virtual models can simulate complex biological processes, including drug metabolism, distribution, target engagement, and potential toxicities, providing valuable insights before human exposure.

In oncology specifically, creating DTs confers the capacity to radically transform cancer research and development. Table 1 summarizes the general contributions and challenges of DTs in clinical trials design. These aspects are supplemented with considerations beyond clinical trials. DTs can reproduce patient and tumor characteristics through accessible computational platforms that enable rapid hypothesis testing and treatment optimization. Cancer is heterogeneous nature, with its diverse genetic, epigenetic, and microenvironmental factors and makes it particularly suited for DT modeling approaches that can capture this complexity.

**Table 1:** Contributions and challenges of DTs in clinical trial design and beyond.

DTs and design of clinical trials	
Contributions [19–21]	Challenges
Refinement of inclusion criteria. Prediction of expected outcomes across different endpoints. Exploration of various endpoints to determine the most relevant ones. Assessing the required number of subjects under different scenarios (e.g., evaluating long-term benefits). Evaluating the impact of interim analysis timing on trial results.	Incorrect interactions or suboptimal parameterization leading to inaccurate predictions. Difficulty in refining models for novel therapies with restricted data. Reliance on imperfect or simplified biological assumptions.
**DTs beyond clinical trials**	
**Contributions [22–24]**	**Challenges**
Extrapolation of post-study (long-term) data Support for medical-economic modeling Extension of findings to specific populations	Requirement for sufficient and high-quality data to define model parameters and generate credible scenarios

The functioning of DT-based these platforms involves three distinct steps with globally: (i) *Data collection and integration*: Data is gathered from diverse sources such as patient files (electronic health records, genomic profiles, imaging studies, laboratory results) and industry information repositories. This multi-modal data forms the foundation of digital representation. (ii) *Model development and diversification*. The collected data is processed and diversified by applying learning models that allow for the analysis of fewer patients with more variables. These algorithms identify patterns and relationships that would be impossible to discern through conventional statistical methods. (iii) *Validation and refinement*: The final critical step is human validation, where synthetic data and model predictions are rigorously compared with expert experience and experimental outcomes. This validation process ensures DT accurately reflects biological reality. More realistically, although pharmaceutical companies and research teams are currently exploring in depth the creation of DTs with concrete applications, it remains that the development of a fully completed, comprehensive patient DT still constitutes a future objective to be reached [25].

Leading platforms for DT development are currently Jinkō, Dassault Systèmes “3D-EXPERIENCE,” Unlearn, Philips “HealthSuite” and Siemens “Healthineers” Digital Twin Technology supporting applications such as personalized medicine, predictive analytics, clinical trials, and treatment planning [26]. In addition, companies Deeplife and Turbine propose commercial solutions incorporating AI to elaborate DTs of human cells covering multi-omics data and incorporating pathway analysis, allowing in the final simulation of drug response [27, 28].

However, an inherent uncertainty associated with biological systems makes that DTs must be continuously refined, modified, or discarded through continuous evaluation [29]. Model performance is generally lessened on task variants that deviate from the initial assumptions [30]. This limitation results from the continuous advances in basic science research, creating a gap between the capacity to integrate novelty and concrete translational and clinical applications. Thus, a plan of action where DTs would occupy a central place should constitute a closed-loop system ensuring a continuous and increasingly accurate representation of the physical twin [31].

## DTs and preclinical development of anticancer agents

A particularly high degree of sophistication in drug design can come from *in silico* designs, allowing a tridimensional structural drug binding estimation [32]. This innovative strategy can predict the bound protein-ligand conformation in a single step, avoiding the necessary prior knowledge of the protein’s target pocket. Significantly accelerating the drug discovery pipeline, DTs simulate biological systems with high fidelity, allowing the conduct of virtual experiments before advancing to costly *in vitro* and *in vivo* testing. For instance, among the key mechanisms of multidrug resistance in cancer is the overexpression of ATP-binding cassette (ABC) transporters [33]. These membrane proteins act as efflux pumps that actively export drugs from cancer cells, reducing intracellular drug concentrations and diminishing therapeutic efficacy. Interestingly, virtual screening and AI-based applications can better predict the drug-binging characteristics of ABC transporters, thus offering a higher potential for detecting resistance mechanisms and developing strategies to overcome them [34]. This includes designing ABC transporter inhibitors or creating drug modifications that evade efflux, ultimately improving treatment outcomes in resistant cancers. Thus, AI-driven DTs have emerged as a versatile tool impacting the early stages of anticancer drug discovery and development by identifying therapeutic targets and evaluating interactive feedback between drugs and their cellular targets.

One can thus dispose of integrative multifactorial models, comparable to DTs, which can create original novel molecules that are simultaneously evaluated for their chemical validity, their synthetic accessibility, and in fine for their drug-like properties [35]. These comprehensive frameworks ensure that computational drug design produces candidates with favorable physicochemical, pharmacokinetic, and toxicological profiles before substantial resources are invested in their synthesis and testing. Integrating DTs with high-throughput experimental validation creates a powerful iterative workflow where computational predictions inform laboratory tests and experimental results refine digital models. Functional cancer cell models are now at our disposal, resulting from mathematical descriptions of cellular and intercellular mechanisms of the biological systems [36]. These models can generate DT depicting the real system at different levels of abstraction. On these bases, developing new applications for targeted cancer treatment becomes conceivable, as underlined by Carracedo-Reboredo and coworkers [37]. Interestingly, a recent report by Klomp and coworkers [38] provides a comprehensive molecular portrait of the ERK-regulated phosphoproteome, including 2123 ERK-dependent phosphoproteins, thus offering a wide range of opportunities for original therapeutic tool development. Recent advances in creating computational models have led to describing pathophysiological situations, which can be represented as a molecular interactome, including hubs and master regulators, which play a central role in developing the pathology [39]. It is anticipated that the generation of molecular maps can produce AI models that reveal hidden cancer vulnerabilities [40]. On such bases, DTs promises to revolutionize pharmaceutical development, especially for drug discovery and validation [18]. Many drug efficacy datasets obtained on cell lines have been made available, thus allowing an *in silico* approach to be developed to predict drug activity [41]. Particularly interesting is the possibility of applying a multidimensional strategy integrating chemical information with genomics, transcriptomics, and proteomics [42]. Cellular models, by simulating different individual sensitivities to treatment, could be complemented or even totally replaced by the DT approach in the near future [43]. For instance, among the currently relatively rare applications of DT in a preclinical setting, there is to underline the possibility to predict the cytotoxic efficacy for more than 17,000 screened molecules among 59 human cancer cell lines in the NCI-60 human tumor Cell Lines Screen [44]. Of importance was the prediction of cancer drug synergy, which was also made possible through the application of multidimensional strategies [45]. Moreover, through the integration of 6 omics data types from 10 cancer categories with biological support from a combined resource of cell lines and human tissue, Savage and coworkers [46] were able to create a comprehensive and expanded set of proteins and peptide targets covering several therapeutic modalities. On the other hand, a promising development of a DT-based bioprocess DT has been reported [47]. This approach is relevant to accurately predicting cell culture profiles and creating virtual replicas of manufacturing conditions, allowing, in particular, optimal production of recombinant therapeutic proteins dedicated to the targeted therapy of cancer.

It thus appears that functional and non-phenomenological DTs embracing the field from single cells to cell–cell interactions to 3D tissue models can now open new areas for cancer research and drug development by adopting drug laboratory strategies that will complement established *in vitro* and *in vivo* preclinical models [36]. Last but not least, DTs of animal models can represent alternative solutions to animal welfare, possibly predicting the toxicity profiles of novel drugs [13].

## DTs and clinical development of anticancer agents

The wider domain of DT application in general health and cancer care, mainly, lies in patient-specific representations [39]. These virtual models of patients can be created as substitutes for real patients, capturing individual biological characteristics, disease states, and potential treatment responses with increasing fidelity. This personalized approach represents a significant paradigm shift from traditional population-based methodologies to truly individualized cancer management (Table 2).

**Table 2:** Applications of DTs in cancer treatment.

Field	DT application	Description	Main data	Challenges and limitations
**Treatment Outcome Simulation**	**Oncosimulator (14)**	Lattice-based model simulating tumor cell kinetics at super-cellular and tissue scale for tumor growth or treatment conditions.	Accurately simulates changes in tumor size, shape, and position.	Difficulty in capturing tumor dynamics over time, particularly in complex cancers. May oversimplify tumor biology heterogeneity.
	**GBM Model (GLIOMATH) (43)**	Patient-specific mechanical models simulating GBM growth by integrating biochemical and mechanical tumor-microenvironment interactions.	A 99% match to MRI data was observed at 8 months, but the accuracy was limited beyond 10 months.	Difficulty capturing tumor growth. Limited adaptability to long-term changes and imperfect simulation of dynamic tumor environments.
	**CPDT (11)**	Personalized models using large breast cancer datasets to visualize cancer evolution and treatment responses.	Identifies distinct immune patterns for personalized treatment management.	Heavily dependent on the quality of available data and may oversimplify complex biological interactions. Data quality and completeness can lead to biased predictions, failing to accurately reflect the real-world complexities.
	**DITTO (Visual Digital Twin) (50)**	Interactive virtual patient model simulating treatment outcomes over time, using dynamic charts and graphs.	Enhances clinician understanding of treatment trajectories.	Difficulty in simulating all possible real-world scenarios and constraint by precise, current patient data to function effectively.
**Virtual Trials**	**Pembrolizumab Virtual Trial (45)**	Simulated NSCLC trial for patients with PD-L1 TPS ≥ 50%, evaluating outcomes beyond progression.	Predictions aligned with clinical outcomes, suggesting post-progression benefit.	Reliance on virtual datasets may not cover all patient diversity, limiting generalizability.
	**Durvalumab Virtual Trial (46)**	Simulation of Durvalumab efficacy in NSCLC patients using virtual Populations.	Matches real-world outcomes for high PD-L1 expressers in clinical settings	Limited by data variability, some patient responses may not be captured accurately.
	**FLAURA2 and MARIPOSA Virtual Trials (47)**	Virtual simulations using the Jinkō platform to predict outcomes in lung cancer trials.	Accurately predicts hazard ratios and survival, consistent with real-world trials.	Generalizability issues may limit the applicability of virtual trials to real-world patient diversity.
	**Chemotherapy Response Prediction (48)**	DT model using cumulative data from phase II/III randomized trials to optimize chemotherapy regimens.	Improved ORR and survival outcomes, helping optimize chemotherapy regimens.	Relying on historical data can limit the adaptability to novel tumor findings characteristics.
	**FarrSight-Twin Technology (48)**	Integrates clinical and genetic data across multiple cancer types to optimize treatment regimens and simulate outcomes.	Patients matched with DT-based predictions exhibited better responses and survival outcomes. The approach enhanced single-arm trials by using patients as their own synthetic controls.	Requires comprehensive data integration, limited in reflecting the full complexity of each patient’s unique cancer biology.
**Real-time Virtual Patient accrual**	**I-SPY 2 Trial (4)**	Real-time adjustments of breast cancer treatments based on biomarker-driven classification into subtypes to improve treatment matching.	Improved drug development efficiency, with response rates rising from 19% to 60%.	Resource-intensive, requiring continuous monitoring and large computational resources for wide-scale application.

DITTO, digital twin for interventions and temporal treatment outcomes; DT, digital twin.

The first domain of DT application in cancer treatment may concern the prediction of disease evolution. In this respect, an Oncosimulator, a lattice-based, discrete-event, discrete-state model, can simulate tumor cell kinetics at a super-cellular and tissue scale under free-growth or treatment conditions [14]. This sophisticated computational platform integrates multiscale data—from molecular alterations (genomics, proteomics, metabolomics) to macroscopic tumor characteristics—to forecast how cancers progress over time with or without intervention. As a part of the CHIC (Computational Horizons in Cancer) platform, there were two reported applications based on Oncosimulators (Table 2): Wilms Tumour Oncosimulator for preoperative chemotherapy response modeling in nephroblastoma and lung Oncosimulator for external beam radiotherapy response modeling in lung tumors (NSCLC) [14]. The primary objective of these approaches was to evaluate how accurately these models can predict changes in tumor size, shape, and position in response to therapy (for Wilms Tumour) or natural growth (for NSCLC). The model effectively captured distinct growth and shrinkage patterns for each cancer type. The CHIC platform thus offers real-time adjustments that align closely with each patient’s unique disease profile [4, 14]. Data were anonymized within the CHIC European Commission-funded program [14]. Another complex and aggressive tumor, Glioblastoma Multiforme (GBM), was also the subject of a prospective observational trial: GLIOMATH (Table 2). This application develops patient-specific mechanical models simulating the growth of GBM by integrating biochemical and mechanical interactions between the tumor and its microenvironment. Personalized virtual tumor models were created using MRI data from individual patients to track tumor progression. In a concrete case of a 55-year-old patient with right temporal GBM, the model accurately predicted tumor growth, with a 99% match to magnetic resonance imagery data at 8 months and a high Jaccard index of 0.8504. However, at 10 months, the model had difficulty capturing the tumor’s complex dynamics, significantly as it infiltrated areas altered by surgery [48]. This observation underlines a limit of the model, with difficulties in matching the reality of tumor evolution over time.

Creating Cancer Patient Digital Twins (CPDT) is an ambitious project of the University of Massachusetts where the investigators started by analyzing data from 1218 primary breast tumors from the TCGA Breast Cancer dataset and 1904 tumors from the Molecular Taxonomy of Breast Cancer International Consortium (METABRIC) [11] (Table 2). Then, they developed a data-driven ordinary differential equation model for human breast tumors and created CPDT to offer a patient-specific experience that visualizes cancer evolution based on individual disease characteristics [11]. Of importance, the investigators identified five distinct immune patterns of human breast tumors, offering concrete perspectives for managing and optimizing treatment. Another concrete application of DT is to compare data from virtual trials with those obtained from conventional trials to validate their reliability. For example, a virtual trial was developed by Socinski and coworkers where response rates for pembrolizumab across treatment lines and patient demographics were informed by baseline covariates [49] (Table 2). The model investigated the capacity of pembrolizumab treatment beyond progression in patients with NSCLC with PD-L1 TPS ≥ 50%. The findings indicated that patients experiencing progression in non-target lesions under pembrolizumab would likely continue benefiting from treatment [50]. Following this model, *in silico* clinical trial simulation of PD-L1 inhibition (durvalumab) used virtual patients, whose data closely matched real patient information, to predict checkpoint inhibitor efficacy in stage III NSCLC (Table 2). Interestingly, the model’s predictions aligned with clinically observed confidence intervals, confirming that patients with high PD-L1 expression had a higher overall response rate (ORR) than those with low PD-L1 expression [51].

Still, in NSCLC, trial protocols for FLAURA2 and MARIPOSA-two conventional clinical trials were simulated using the Jinkō platform [52]. By integrating a computational model, a virtual patient cohort, and the specific protocols of each trial, investigators conducted virtual trials that mirrored the durations of actual trials: 33 months for FLAURA2 and 32 months for MARIPOSA. Remarkably, the results demonstrated that in silico predictions were highly consistent with real-world outcomes (Table 2). For instance, the FLAURA2 simulation data closely aligned with actual trial data regarding hazard ratios and median survival times. Similarly, predictions for the osimertinib arm of the MARIPOSA trial were also highly accurate through the DT-based approach [52]. These findings strongly indicate that virtual trials based on mechanistic models can reliably replicate clinical outcomes and contribute to trial design improvements. The capacity of DT models to accurately predict individual responses to specific chemotherapy treatments was also reported (Table 2). Thus, cumulative data from eight phase II or III randomized trials comparing chemotherapy regimens were analyzed in one study. In these trials, teams were unblinded for four studies and blinded for the final four simulations. The DT model successfully predicted the log odds ratio for ORR in each treatment arm and demonstrated strong alignment with actual trial outcomes [53]. The authors applied the model to identify optimal standard-of-care treatments for various patient cohorts. A key component was the FarrSight-Twin technology, which integrates clinical and genetic data to simulate responses across multiple cancer types, demonstrating improvements in ORR and survival compared to standard treatments [53, 54]. The findings revealed that patients matched with DT model-based predictions showed better responses and survival outcomes. This DT-based approach not only tailored treatments based on clinical and genetic insights but also enhanced single-arm clinical trials by enabling patients to serve as their own synthetic controls, thereby improving trial interpretation and advancing the personalization of cancer therapies [53]. DITTO appears as a DT and visual computing system designed to deliver risk-based treatment for head and neck cancer patients [55]. In this study, the authors disposed of a dataset of 600 patients with oropharynx cancer, modeling 19 different outcomes and transition state variables. DITTO was prospectively clinically validated, showing the reliability of the DT-based treatment setting approach. This validation represents a significant milestone in DT implementation, demonstrating that computational predictions can successfully translate to meaningful clinical outcomes in complex cancer types. At least two levels of applications concern the implication of DTs in clinical trial development for cancer treatment. First, DTs can simulate comparator arms and thus permit an earlier assessment of clinical efficacy for an emerging new drug [13]. Another interesting contribution of DTs in clinical research is to permit a real-time patient adjustment for an ongoing trial. For instance, the I-SPY 2 trial allowed real-time adjustments in treating breast cancer patients based on ongoing results [4] (Table 2). By utilizing a biomarker-driven strategy to classify breast cancer into ten subtypes, I-SPY 2 enabled a more precise treatment matching. The design improved drug development efficiency, having evaluated 24 agents over 12 years, with response rates increasing from 19% to approximately 60%. Focusing specifically on the neoadjuvant setting, the model offered advantages such as real-time treatment response monitoring using pathologic complete response as a primary endpoint. Globally, the application has resulted in identifying optimal treatment strategies at the individual level and rapidly predicting which patients will benefit from a particular regimen [4].

## Current limitations of DTs and future directions (Fig. 1)

**Figure 1:**
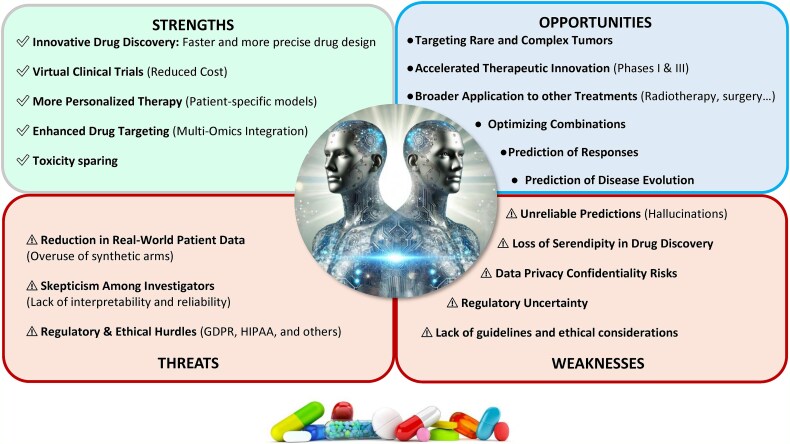
Digital twins in cancer treatment development: SWOT analysis.

It is clear that the DT model can significantly reduce the cost and time of clinical trials by simulating patients’ biological processes, responses, and adverse reactions and refining treatment doses without exposing patients to unnecessary risks. DTs can also enhance real-time treatment assessments for more individualized approaches that outpace traditional trial limitations [4, 14]. It is essential to incorporate synthetic patients, as it may increase trial efficiency by amplifying the sample size, as recently reported in the management of acute myeloid leukemia [56].

However, the approach involving the application of DTs is not the only method for improving clinical research by increasing sample size. A Bayesian adaptive synthetic-control design for phase II clinical trials has also been reported [57]. The model exploits historical control data and addresses the problem of bias when comparing trial results to historical data. The proposed design led to a similar power to that of randomized controlled trials but necessitated a substantially smaller sample size. Alternatively, a well-established method for using historical control data to build bias-corrected estimators is based on propensity score matching [58]. Recently, propensity score matching has led to several new oncologic drug approvals by the FDA, such as palbociclib in HR+/HER2− metastatic breast cancer using synthetic-control data [59]. Both propensity score-based methods and Bayesian adaptive synthetic-control designs are not exempt from limitations. The main one is the possible presence of unmeasured confounders [57]. Clearly, using DTs can circumvent the potential issue linked to the number of parameters in the model since the AI-based approach with DTs can encompass an unlimited number of factors, allowing DT shaping [58]. Conversely, the use of DTs in anticancer drug development is not without limitations. At a preclinical stage for drug discovery, it must be recalled that historical evidence exists that many major anticancer drugs like cisplatin and analogs, in opposition to what AI-based DT could rationally generate, have been discovered fortuitously through the so-called phenomenon of serendipity [9]. Thus, with the widespread adoption of DT-based tools for new drug identification, there is a risk that human creativity, imagination, and divergence from AI logic could be discouraged [60]. As recently highlighted by Gao *et al*. [61], a limitation in developing agent systems like DTs is their propensity to produce unreliable predictions and non-factual information, reasoning errors, and systematic biases. To this list, one can add a lack of interpretability [62], which can introduce skepticism among investigators, thus down-tuning inflated expectations about the promise of DT performances in anticancer drug development. Another limitation of applying DT in clinical trial development is the potential depletion of real-world patient-related information. This issue could arise from an increased reliance on synthetic arms in clinical trials where anterior data are rearranged to create new bases for the comparisons. In this respect, DT applications could be prioritized in clinical trials where they enable real-time adjustments by incorporating digital patients, thereby optimizing trial efficiency through an expanded sample size. It is important to emphasize that DT models must represent populations from diverse ethnic, socio-economic, and geographic backgrounds to ensure their applicability across various demographic groups. Without such diversity, DT simulations are at risk of producing biased or ineffective results, particularly in cancer, where genetic and environmental factors significantly impact disease progression and treatment response. Integrating data from both high- and low-income regions with varying levels of healthcare digitalization can help mitigate these risks, fostering more equitable and effective healthcare solutions [50, 51, 63].

Moreover, privacy and regulatory challenges are equally crucial in DT applications. They demand access to vast amounts of personal health data, necessitating strict adherence to regulations like the GDPR (General Data Protection Regulation) in Europe and HIPAA (Health Insurance Portability and Accountability Act) in the United States, ensuring privacy and security [4, 50, 64].

Frameworks such as the European AI Act and the EMA’s guidelines are crucial in standardizing DT applications, ensuring that models meet rigorous standards of accuracy, privacy, and ethical compliance. These regulatory measures are vital for protecting patient rights and supporting DT’s trustworthiness and reliability [51, 63, 65].

The future of DT applications in anticancer drug development appears promising, as they integrate both preclinical and clinical aspects, offering tailored solutions to overcome current challenges such as high costs and prolonged data generation times. Not in the scope of the present position paper, there is expanding research in predictive radiation oncology which can be anticipated, mainly through the use of DTs. This approach aims to tackle a significant challenge: the long time required for late toxicity, which complicates clinical research in radiation oncology [66].

## Conclusion

One can reasonably estimate that DTs may significantly transform drug discovery and development settings covering a broad spectrum, from the modeling of cell cultures to animal testing to, ultimately, reaching patients. Clinical trial design and applications can be markedly impacted by the transformative implementation of DTs, which generates clear benefits while raising important concerns (Table 1). It must be recognized that the presently covered DT field is still underway with more undisputable capacities to undertake than concretely exploitable achievements. The technology promises substantial improvements in efficiency and personalization, but practical implementation faces challenges related to data quality, model validation, and regulatory acceptance. Above all, DTs represent a perfect illustration of what AI can generate in progress in new drug development, particularly in the cancer area. They should motivate a wave of initiatives both on the preclinical and clinical sides. The achievement of these objectives will require an active collaboration among a diverse group of specialists, including mathematicians, biologists, and physicians [11]. This interdisciplinary approach is essential for developing DTs that accurately capture the complexity of cancer biology while remaining computationally tractable and clinically relevant. The current absence of regulations addressing validation requirements, ethical considerations, and implementation standards may be an obstacle to the widespread adoption of DTs in preclinical and clinical trials. Clearly, all stakeholders in the DT ecosystem are eagerly awaiting stronger engagement from healthcare agencies to establish clear guidelines, ensuring that DTs can become a major breakthrough in all aspects of cancer drug development.

**Key Points**

- **New drug discovery:** Digital twins can predict the cytotoxic efficacy of several thousand screened molecules based on pharmacogenomic databases from the NCI-60 human tumor cell lines panel. This computational approach enables high-throughput virtual screening that identifies promising candidates with greater precision, reducing resource expenditure on compounds unlikely to demonstrate clinical efficacy.
- **Accelerated drug development:** By conducting “virtual” clinical trials on digital twins, researchers can quickly test new drugs and treatment combinations, reducing the time and cost of traditional trials. This can lead to faster development of new therapies while minimizing patient exposure to potentially ineffective or harmful treatments. The virtual testing environment allows for extensive exploration of dosing regimens, scheduling, and combination strategies before human implementation.
- **Improved trial design:** Digital twins have enabled a significant breakthrough: the ability to perform real-time patient adjustments during ongoing clinical trials. This adaptive approach allows investigators to optimize treatment parameters based on accumulating data, potentially improving outcomes for trial participants while generating more informative results for subsequent development decisions. Additionally, DTs can enhance patient stratification, identifying subpopulations most likely to benefit from specific interventions.
